# Polymorphism of Interleukin 1B May Modulate the Risk of Ischemic Stroke in Polish Patients

**DOI:** 10.3390/medicina55090558

**Published:** 2019-09-02

**Authors:** Iwona Gorący, Mariusz Kaczmarczyk, Andrzej Ciechanowicz, Klaudyna Lewandowska, Paweł Jakubiszyn, Oksana Bodnar, Bartosz Kopijek, Andrzej Brodkiewicz, Lech Cyryłowski

**Affiliations:** 1 Department of Clinical and Molecular Biochemistry, Pomeranian Medical University, 70-111 Szczecin, Poland; 2 Department of General and Dental Radiology, Pomeranian Medical University, 70-111 Szczecin, Poland; 3 Department of Pediatrics, Child Nephrology, Dialysotherapy and Management of Acute Poisoning, Pomeranian Medical University, 71-899 Szczecin, Poland; 4 Department of Intervention Radiology, Pomeranian Medical University, 70-111 Szczecin, Poland

**Keywords:** *IL1B*, *IL1RN*, polymorphism, stroke, inflammation

## Abstract

*Background and Objectives*: Inflammation plays a crucial role in the pathophysiology of ischemic stroke (IS). Interleukin-1B and interleukin-1 receptor antagonists are key factors in inflammatory processes. Aims: The aims of our study were to evaluate the relationship between genetic variation in interleukin-1B (*IL1B*) rs1143627 and interleukin-1 receptor antagonist (*IL1RN*) variable-number-tandem-repeats (VNTR), and overall IS and subtype prevalence rates. *Materials and Methods:* The analysis included 147 hospitalized Polish patients with IS diagnosed using conventional criteria. The control group consisted of 119 healthy subjects. Genotypes were determined by polymerase chain reaction. *Results:* A significant association between rs1143627 and stroke was found. The -31C *IL1B* polymorphism showed an association with overall IS, OR = 2.30 (1.36–3.87) *p* = 0.020. An association was also detected for LVI (large vessel infarction) subtypes of stroke. After risk factor adjustment (age, diabetes mellitus, dyslipidemia), the C allele was found to be an independent risk factor for LVI, OR = 1.99 (1.05–3.79) *p* = 0.036. Significant association was not observed between *IL1RN* alleles and IS. *Conclusions:* Our results suggest that the C allele of *IL1B* rs1143627 may be associated with susceptibility to overall IS and LVI subtypes of stroke in the Polish population.

## 1. Introduction

Ischemic stroke (IS) is a multifactor disease, resulting from classical and genetic risk factors and their interactions. Accumulating evidence supports a critical role of inflammation in the pathogenesis of IS. Interleukin-1 (IL1) is one of the key pro-inflammatory cytokines which plays a key role in this inflammatory process. The IL1 family consists of IL1A, IL1B and one antagonist cytokine, the IL1 receptor antagonist (IL1RA) [1,2]. IL1A and IL1B are inflammatory factors produced by different cell types in response to various stimuli. They affect the endothelial cells, including the induction of adhesion molecules and prothrombotic effects, while the naturally occurring competitive IL1RA may antagonize the immune response. Disturbed balance in the action of IL1A, IL1B and IL1RA leads to the development and progression of atherosclerosis. In fact, earlier studies have shown that increased levels of inflammatory cytokines are associated with vascular ischemic disease [3,4,5].

The IL1 gene cluster, with loci on chromosome 2, encompasses the *IL1A*, *IL1B*, and *IL1RN* genes. The polymorphisms in this *IL1* gene cluster, including *IL1B* and *IL1RN* (interleukin-1 receptor antagonist, encoding IL1RA), have been commonly studied and appeared to be associated with plasma levels of IL1B and ILRA [6,7].

In experimental studies, several independent groups have reported an early increase in IL1 expression in response to cerebral ischemia in rodents [8,9]. It has also been demonstrated in wild-type (WT) and knock-out IL1RI (IL1RI KO) mice that IL1 may exacerbate ischemic brain injury independently of IL1RI, which suggests the existence of an additional IL1 receptor or receptors in the brain [10]. Currently, IL1 polymorphism is considered to be an independent risk factor for IS development, although some studies have not confirmed this relationship.

The polymorphism in intron 2 of the interleukin receptor antagonist gene (*IL1RN*) is caused by the variable copy number of an 86-bp sequence. The most common allele, allele 1 (IL1RN*1), contains two repeats. The alleles 2, 3, 4, and 5 have two, five, three, and six repeats, respectively [11]. The IL1RN*2 allele of the variable number tandem repeat (VNTR) of *IL1RN* has been reported to be associated with increased ILRA production, which naturally downregulates the immune response [12]. However, some studies have shown that IL1RN*2 is associated with decreased IL1RA production. IL1B influences the endothelium, including the induction of adhesion molecules and procoagulant activity. Thus, the IL1/IL1RA balance may modulate inflammation processes which may contribute to the pathogenesis of ischemic stroke. A pro-inflammatory profile comprising SNPs in gene encoding regions of *IL1B* and *IL1RN* was further reported to confer an increased risk of atherosclerosis development [13,14,15] and some studies have reported that *IL1B* and *IL1RN* polymorphisms are associated with genetic risk of IS [16]. However, other studies on different populations did not confirm this and the association remains controversial [17,18].

Ischemic stroke is a disease with devastating consequences, which is why we are still looking for markers enabling early diagnosis. Currently, many risk factors are known for the development of stroke. However, our knowledge concerning the genes which promote the development of stroke is still limited. Our study attempts to explain the role of the genetic variants of *IL1B* (C(-31)T)and *IL1RN*, considered to be key factors regulating inflammatory processes in the development of ischemic stroke. Therefore, we have investigated the possible association between genetic variation in *IL1B* rs1143627 and *IL1RN* VNTR with overall IS and subtypes of IS classified by TOAST (see Materials and Methods) in the Polish population.

## 2. Materials and Methods

### 2.1. The Study Group

A total of 147 unrelated patients (80 males and 67 females) were admitted to hospital because of acute brain ischemic stroke (IS): Diagnosed using conventional criteria, including rapidly developed focal or global disturbance of cerebral function lasting more than 24 h, without CT signs of a hemorrhagic lesion in the brain. The study group was from a homogeneous Polish population. All 147 patients underwent clinical scrutiny, investigation of medical history and family anamnesis, evaluation of vascular risk factors, general physical and neurological examinations, routine biochemical analyses, ECG (electrocardiography), and computed tomography (CT) of the brain, within two days of onset.

Data from risk factors were recorded, including arterial hypertension (HT, defined as systolic blood pressure exceeding 140 mmHg or diastolic blood pressure greater than 90 mmHg or previous diagnosis), body mass index (BMI, calculated as weight/height^2^), and diabetes (DM, previously diagnosed or a fasting plasma glucose concentration > 7.8 mL/L). Patients were classified as “current smokers” if they reported smoking more than five cigarettes per day. Routine biochemical analyses were done including fasting blood glucose, total cholesterol, HDL cholesterol and triglycerides, liver, and kidney function tests.

The study population was divided according to the TOAST classification [19], which identified five causes of ischemic cerebral infarction: (1) Large artery atherosclerosis (LVI—large-vessel infarction) in 71 patients (46.7%), (2) small-vessel occlusion (SVI—small vessel infarction) in 40 patients (26.3%), (3) cardioembolism (CEI—cardioembolic infarction) in 24 patients (15.8%), (4) stroke of other determined etiology (e.g., non-atherosclerotic artery disease) in no patients, and (5) stroke of unknown etiology in 17 patients (11.2%).

The control group consisted of 119 subjects (65 men, 52 women) who reported non-specific chest complaints and were diagnosed in regard to CAD. They underwent coronary angiography which detected no lesions in coronary arteries. A medical examination ruled out IS and other atherosclerotic diseases as well as a history of ischemic, hemorrhagic, and other brain diseases. The protocol of the study was approved by the Pomeranian Medical University Ethics Committee (nr BN-001/119/03/16.03.2003), with formal informed consent signed by all participants.

### 2.2. Genotyping

Genomic DNA was isolated from peripheral blood leukocytes using a commercial kit (QIAamp DNA Mini Kit; Qiagen, Hilden, Germany).

For the analysis of C(-31)T *IL1B* gene polymorphism (rs1143627) a polymerase chain reaction/restriction fragments length polymorphism (PCR/RFLP) method was applied with the following primer pair: Forward: 5′ AgA AgC TTC CAC CAA TAC TC, and reverse: 5′ AgC ACC TAg TTg TAA ggA Ag (TIB MOL BIOL, Poznań, Poland). Amplification was performed in volumes of 10 μL containing 40 ng genomic DNA, 0.1 μL of each primer, 5 μL 2xPCR Master Mix (Fermentas, Vilnius, Lithuania). The reactions were run under the following conditions: Denaturation (94 °C, 5 min), annealing (56 °C, 40 s), and extension (72 °C, 8 min). Thirty-five cycles were performed using a Mastercycler gradient machine (Eppendorf, Hamburg, Germany). The resulting product (234 bp) was digested with the Alu I restriction enzyme (MBI Fermentas, Vilnius, Lithuania), and the digestion products were separated in 4% agarose gels. The polymorphic region within intron 2 of the *IL1RN* gene was amplified using polymerase chain reaction (PCR). Genomic DNA (20 ng) served as a template in the 10 μL PCR reaction. This reaction contained the following components: 0.1 μL of each forward primer: 5′ CCC CTC AgC AAC ACT CC, and reverse primer: 5′ ggT CAg AAg ggC AgA gA (TIB MOL BIOL, Berlin, Germany); 5 μL 2xPCR Master Mix (Fermentas, Vilnius, Lithuania) the reaction was performed using standard settings: Denaturation (94 °C, 5 min), annealing (58 °C, 1 min) and extension (72 °C, 8 min), 36 cycles performed using a Mastercycler gradient machine (Eppendorf, Germany). The sizes of amplified products were determined by electrophoresis on 3% agarose gels.

### 2.3. Statistical Analysis

Statistical analysis was conducted with the R statistical platform (http://cran.r-project.org) using the package SNPassoc (SNPs-based whole-genome association studies. R package version 1.9-2. https://CRAN.R-project.org/package=SNPassoc). In the analysis of single SNPs, multiple inheritance models were used: Co-dominant, dominant, and recessive. Analysis of gene–gene interactions was carried out for the dominant and recessive models. Inheritance models were created with respect to minor alleles. The significance of interactions was calculated by comparing two models with and without the interaction term, using likelihood ratio tests. *p* < 0.05 was considered statistically significant.

## 3. Results

Demographic characteristics of the IS and control groups, risk factors and TOAST classifications in stroke cases are shown in Table 1. All characteristics did not differ between the two groups except for age (66.9 ± 12.1 vs. 56.8 ± 9.8, *p* < 0.0001) and the frequencies of diabetes mellitus (27% vs. 13%, *p* = 0.004) and dyslipidemia (16%/84% vs. 76%/24%, *p* < 0.0001).

### 3.1. Association between Overall IS and Genetic Variation in IL1B and IL1RN

The genotype distributions were in Hardy–Weinberg equilibrium for *IL1B* (all individuals *p* = 0.081, cases *p* = 0.741, control group *p* = 0.073) and *IL1RN* (all individuals *p* = 0.794, cases *p* = 0.867, control group *p* = 0.675). Observed genotype frequencies for the *IL-1B*:C(-31)T polymorphism (rs1143627) were: 33.3% T/T (*n* = 88), 43.9% C/T (*n* = 116), 22.7% C/C (*n* = 60) and for alleles: 55.3% T and 44.7% C. The genotype frequencies for the *IL1RN* were 39.0% 1/1 (*n* = 103), 46.2% 1/2 (*n* = 122), 14.8% 2/2 (*n* = 39) and for alleles: 62.1% 1 and 37.9% 2. The *IL1RN* alleles 3 and 4 were rare and their frequencies did not significantly differ between the IS patients and the control group (1.9% vs. 1.6%, *p* = 0.765 for allele 3 and 0.3% vs. 1.2%, *p* = 0.122 for allele 4). Baseline characteristics of the study group are shown in Table 1. The results of tests of association of the *IL1RN* and *IL1B* polymorphisms with stroke are summarized in Table 2 and Table 3.

The results of tests for association of the *IL1RN* and *IL1B* polymorphisms with stroke are summarized in Table 2 and Table 3. In multivariable modeling, including covariates (age, diabetes mellitus, dyslipidemia), the association of the *ILRN* with stroke was insignificant (Table 2). For the *IL1B* polymorphism, the association under codominant and recessive models was insignificant after adjustment for covariates (age, diabetes mellitus, dyslipidemia), however with the dominant model, the risk of stroke with CT-CC was 2.3 higher than for TT homozygotes (2.30 (1.36–3.87); *p* = 0.020) (Table 3).

**Figure 1:**
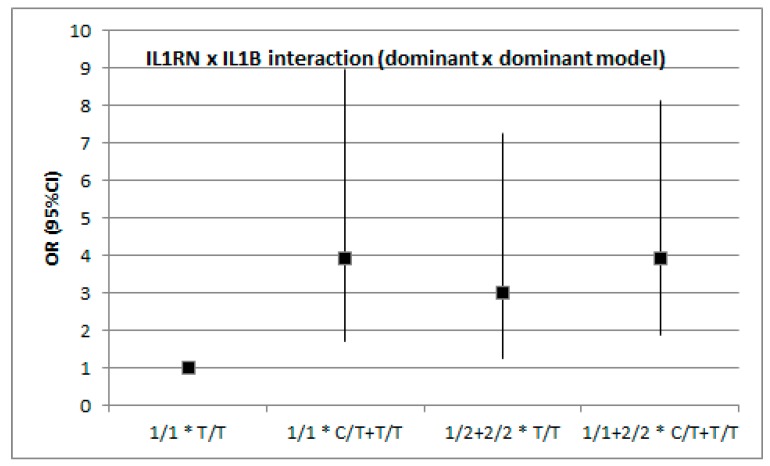
*IL1RN* × *IL1B* interaction (dominant × dominant model). Raw *p* = 0.046, adjusted *p* = 0.232 (age, diabetes mellitus, dyslipidemia status).

**Figure 2:**
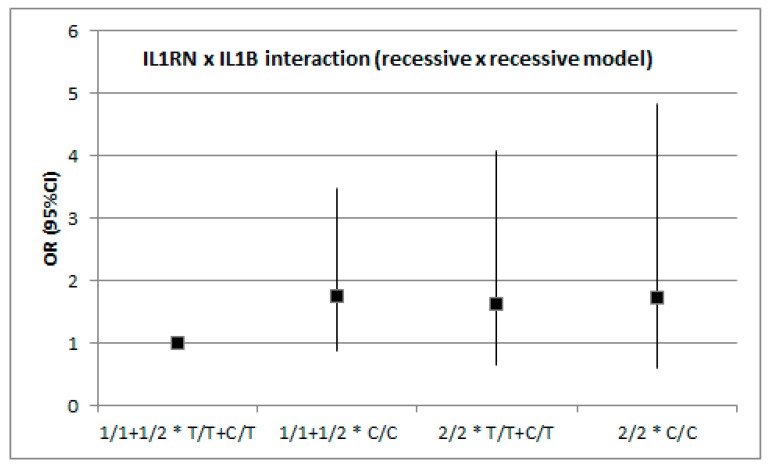
*IL1RN* × *IL1B* interaction (recessive × recessive model). Raw *p* = 0.502, adjusted *p* = 0.910 (age, diabetes mellitus, dyslipidemia status).

In addition to single-locus analyses, we investigated whether the two genes interacted with respect to the modification of stroke risk (Figure 1 and Figure 2). The analysis was conducted with the assumption of dominant or recessive models for each polymorphism and no evidence was found of gene–gene interaction with respect to IS risk. Although the raw *p* value for the dominant x dominant model was 0.046, it turned out to be non-significant after adjustment for covariates.

### 3.2. Association between IS and Genetic Variation in IL1B and IL1RN by Stroke Subtype (TOAST)

Each stroke subtype, i.e., CEI, SVI, LVI of the TOAST classification, was compared with control subjects (Table 4 and Table 5). No significant associations were found between *IL1RN* and stroke under codominant, dominant, and recessive models by stroke subtype according to TOAST classification (Table 4). For the *IL1B* gene, the carriers of the C allele were significantly overrepresented in LVI subtypes compared with controls with (1.99 (1.05–3.79), *p* = 0.036) (Table 5). For the other subtypes, we did not find any significant correlations (Table 5).

## 4. Discussion

Ischemic stroke is a disease of complex etiology, and it is generally accepted that both environmental and genetic factors play a crucial role in the development of the disease. Although there are many studies which have indicated that inflammatory cytokines and their genetic polymorphisms play an important role in the pathogenesis of IS, the results are still controversial. In this study, two important polymorphisms of the IL1 cluster were investigated for their association with stroke. We have not shown any connection between genetic variants of *ILRN* VNTR with overall stroke or subtypes. This is in line with previous studies on IS [20]. In contrast some studies, have reported an association of the *IL1RN* with stroke [21]. This inconsistency could be explained with the rather relatively small size of study groups than differences between populations. However, we have shown that the *IL1B*:T(-31)C polymorphism is independently associated with overall IS and subtype of IS in the homogeneous Polish population.

For the *IL1B*:C(-31)T polymorphism, we found that carriers of the C allele were associated with a higher risk of overall stroke. Moreover, we found that CT/CC genotypes can increase the risk of subtypes of IS. The relationship of *IL1B* polymorphism and stroke has been examined in several previous studies and our results remain in line with those presented so far. Extensive studies on the *IL1B* polymorphism at position -511 have indicated that *IL1B:*-511T carriers had higher levels of IL1B than *IL1B*:-511C and were associated with increased risk of IS [22]. It is also worth mentioning the study by Iacoviello et al. [23] which is the main source of research heterogeneity because this study reported that TT homozygotes of *IL1B*:-511 are associated with a decreased risk of IS. However, patients in this study were relatively young. Thus, the association between *IL1B* polymorphism and IL1B production still remains controversial. A recent study has documented that IL1B mRNA was increased in the TT genotype [24] whereas Hall et al. [25] showed a 2–3-fold increase in IL1B protein secretion in subjects with the T allele at -511 and the C allele at -31.

We found no interaction between *IL1RN* and *IL1B* concerning IS risk assuming the dominant x dominant and recessive x recessive models. The gene–gene interaction was analyzed using a linear model in which only two (and the same) inheritance patterns for each locus were considered. This approach could possibly have less power as compared with a non-parametric and model-free multifactor dimensionality reduction method that has been shown to have reasonable power to detect epistasis [26].

The polymorphism of *IL1B* at -31 is tightly linked with the polymorphism at -511. However, it is unclear whether the C or T allele of *IL1B* -31 is associated with high expression. We have not measured the plasma IL1B and IL1RA levels and this can be considered as a limitation of our study. It is worth emphasizing that genetic variations of *IL1B* -31 and von Willebrand factor are associated with the recanalization rate of fibrinolysis with tissue-type plasminogen activator, and thus with treatment efficacy [27]. As the authors report, the mechanisms by which these SNPs modulate recanalization could be related to homeostasis modulation by modification of coagulation factor activities. Manso et al. [28] also tested the inflammatory genes *IL1B*, interleukin 6 (*IL6*), myeloperoxidase (*MPO*), and *TNF* with stroke susceptibility, and demonstrated that only two SNPs of *IL6* and one *MPO* single-nucleotide polymorphism were significantly associated with stroke risk in their sample. Probably, an observed lesser genetic influence is related to widespread classical risk factors, or lifestyle. Some studies have reported that *IL1B*:C(-31)T and *IL1RN*:VNTR polymorphisms are significantly correlated with the development of CAD, and thus atherosclerosis process [3,29]. However, other studies conducted in different populations have not confirmed this and these associations still remain controversial. In our previous study we showed no association between polymorphisms of *IL1B*:C(-31)T/*IL1RN* (VNTR) or their haplotypes and CAD in the Polish population [30], which may suggest lesser importance of genetic factors in the development of atherosclerotic diseases in our population.

Nevertheless, IL1B is a potent pro-inflammatory cytokine and plays a major role in the development of both inflammation processes and thrombosis. It is hypothesized that in IS development, IL1B is involved in thrombus formation rather than in atherosclerosis progression because IL1 induces tissue factor and plasminogen activator inhibitor type 1 gene expression [31]. However, this cytokine can induce complex biological effects by the regulation of gene expression of multiple cell types [32]. Therefore, more mechanisms should be considered in the development of IS, although both inflammatory and prothrombotic mechanisms seem to play a fundamental role. The formation of a thrombus leading to occlusion of a vessel is the endpoint which is influenced by many factors (genes, hypertension, hyperlipidemia, diabetes mellitus, etc.), and the gene–environment interaction can be of great importance. Thus, the *IL1B* C allele may not be associated directly with IS per se, but could be modulating cytokines in the inflammatory processes that affect the development of atherosclerosis resulting in IS. It has been shown that genetic variants of *IL1B* that predict higher inflammatory phenotypes modify the risk of Lp(a) in mediating long-term cardiovascular events [33]. These results indicate that *IL1B* can modify many pathways involved in the development of atherosclerosis and thus, cardiovascular events. Additionally, some studies have also reported that the risk of stroke increases with the number of high-risk genotypes in proinflammatory gene polymorphisms carried by an individual, thus suggesting that such polymorphisms may act synergistically [34]. We have evaluated genetic variants of only two genes which are crucial in inflammation. IS is a very complicated and extremely complex disease and its pathomechanism is still not fully explained. It is not yet known how many risk factors in the development of atherosclerosis could be modulated by the genetic variants of *IL1B*, which acts as a key regulator in inflammatory processes.

In different populations with varying intensity of classical risk factors, the impact of genetics may be found to have a variable extent. Previous studies have reported that ethnicity or regional locations are very important in the determination of environmental risk factors [35]. One of advantages of the present study is that it included a well-characterized and homogeneous patients’ group (from the northwest region of Poland), but it should be emphasized that our society has strongly expressed classical risk factors for IS. It should be noted that observed differences between the study group and the controls could interfere with the assessment of the role for the *IL1RN* polymorphism in the development of IS. Diabetes, which is a widely recognized risk factor for the development of atherosclerosis, has been more frequent in IS patients, while dyslipidemia has been less frequent. However, the IS patients had been already treated (b-blocker, statin, etc.) which could have downregulated to some extent the inflammatory process agents, while the control group comprised subjects without treatment for this. Moreover, the control group was slightly younger than the IS group, which may have had some impact on the obtained results, and could be considered another limitation of our study. The population included in the study was homogeneous (monoethnic, Polish) and, therefore, our data need to be confirmed in different ethnic groups.

## 5. Conclusions

The complex interplay between genetic backgrounds, clinical and lifestyle factors, and the environment may ultimately lead to the development of stroke. In the present study, we present supporting evidence for a role of the *IL1B* inflammatory gene in stroke susceptibility. Our findings confirm previous genetic observations, highlighting the need for further functional studies, particularly in view of the possible utility of *IL1B* as a diagnostic biomarker for stroke.

## Figures and Tables

**Table 1:** Demographic and risk factors of stroke characteristics.

Characteristic	Cases (*n* = 147)	Control (*n* = 117)	*p*
Age (years)	66.9 ± 12.1	56.8 ± 9.8	<0.0001
BMI (kg/m^2^)	27.6 ± 4.8	26.9 ± 4.2	0.286
Sex (Males)	54% (80)	56% (65)	0.854
Smoking	32% (47)	24% (28)	0.150
Diabetes mellitus	27% (40)	13% (15)	0.004
Hypertension	60% (88)	51% (60)	0.163
Dyslipidemia	16% (23)	76% (89)	<0.0001
**TOAST**			
Large-vessel atherosclerosis	46% (68)		
Cardioembolism	17% (25)		
Small-vessel	26% (38)		
Others	11% (16)		

**Table 2:** An association of the *IL1RN* polymorphism with stroke under codominant, dominant, and recessive model.

Model	Control (*n* = 117)	%	Cases (*n* = 147)	%	OR	95% CI		*p*
Codominant								
1/1	54	46.2	49	33.3	1.00			0.647 *
1/2	49	41.9	73	49.7	1.64	0.97	2.79	
2/2	14	12.0	25	17.0	1.97	0.92	4.21	
Dominant								
1/1	54	46.2	49	33.3	1.00			0.358 *
1/2–2/2	63	53.8	98	66.7	1.71	1.04	2.83	
Recessive								
1/1–1/2	103	88.0	122	83.0	1.00			0.650 *
2/2	14	12.0	25	17.0	1.51	0.75	3.05	

* Adjusted by: age, diabetes mellitus, dyslipidemia.

**Table 3:** An association of the *IL-1B* polymorphism with stroke under codominant, dominant, and recessive model.

Model	Control (*n* = 117)	%	Cases (*n* = 147)	%	OR	95% CI		*p* *
Codominant								
T/T	51	43.6	37	25.2	1.00			0.065 *
C/T	45	38.5	71	48.3	2.17	1.24	3.82	
C/C	21	17.9	39	26.5	2.56	1.30	5.05	
Dominant								
T/T	51	43.6	37	25.2	1.00			0.020 *
C/T-C/C	66	56.4	110	74.8	2.30	1.36	3.87	
Recessive								
T/T-C/T	96	82.1	108	73.5	1.00			0.322 *
C/C	21	17.9	39	26.5	1.65	0.91	3.00	

* Adjusted by: Age, diabetes mellitus, dyslipidemia.

**Table 4:** An association of the *IL1RN* polymorphism with stroke under codominant, dominant, and recessive models by stroke subtype (TOAST classification).

Model	Control (*n* = 117)	CEI (*n* = 25)	OR (95% CI)	*p* *	SVI (*n* = 38)	OR (95% CI)	*p* *	LVI (*n* = 68)	OR (95% CI)	*p* *
Codominant										
1/1	54 (46.2)	6 (24.0)	1.00		13 (34.2)	1.00		25 (36.8)	1.00	
1/2	49 (41.9)	14 (56.0)	2.57 (0.92–7.21)		19 (50.0)	1.61 (0.72–3.60)		34 (50.0)	1.50 (0.79–2.86)	
2/2	14 (12.0)	5 (20.0)	3.21 (0.85–12.09)	0.388	6 (15.8)	1.78 (0.57–5.52)	0.947	9 (13.2)	1.39 (0.53–3.63)	0.974
Dominant										
1/1	54 (46.2)	6 (24.0)	1.00		13 (34.2)	1.00		25 (36.8)	1.00	
1/2–2/2	63 (53.8)	19 (76.0)	2.71 (1.01–7.28)	0.261	25 (65.8)	1.65 (0.77–3.53)	0.854	43 (63.2)	1.47 (0.80–2.72)	0.982
Recessive										
1/1–1/2	103 (88.0)	20 (80.0)	1.00		32 (84.2)	1.00		59 (86.8)	1.00	
2/2	14 (12.0)	5 (20.0)	1.84 (0.60–5.68)	0.695	6 (15.8)	1.38 (0.49–3.88)	0.752	9 (13.2)	1.12 (0.46–2.75)	0.823

* Adjusted by: Age, diabetes mellitus, dyslipidemia.

**Table 5:** An association of the *IL1B* polymorphism with stroke under codominant, dominant, and recessive models by stroke subtype (TOAST classification).

Model	Control (*n* = 117)	CEI (*n* = 25)	OR (95% CI)	*p* *	SVI (*n* = 38)	OR (95% CI)	*p* *	LVI (*n* = 68)	OR (95% CI)	*p* *
Codominant										
T/T	51 (43.6)	5 (20.0)	1.00		8 (21.1)	1.00		19 (27.9)	1.00	
C/T	45 (38.5)	14 (56.0)	3.17 (1.06–9.50)		19 (50.0)	2.69 (1.07–6.74)		33 (48.5)	1.97 (0.99–3.93)	
C/C	21 (17.9)	6 (24.0)	2.91 (0.80–10.60)	0.074	11 (28.9)	3.34 (1.18–9.48)	0.236	16 (23.5)	2.05 (0.89–4.72)	0.069
Dominant										
T/T	51 (43.6)	5 (20.0)	1.00		8 (21.1)	1.00		19 (27.9)	1.00	
C/T-C/C	66 (56.4)	20 (80.0)	3.09 (1.09–8.80)	0.305	30 (78.9)	2.90 (1.22–6.86)	0.106	49 (72.1)	1.99 (1.05–3.79)	0.036
Recessive										
T/T-C/T	96 (82.1)	19 (76.0)	1.00		27 (71.1)	1.00		52 (76.5)	1.00	
C/C	21 (17.9)	6 (24.0)	1.44 (0.51–4.05)	0.225	11 (28.9)	1.86 (0.80–4.34)	0.874	16 (23.5)	1.41 (0.68–2.93)	0.993

* Adjusted by: Age, diabetes mellitus, dyslipidemia.
